# TRIM24 Regulates Adaptation to Glucose Deprivation in Association with Aspartate Accumulation and Impaired AMPK Signaling

**DOI:** 10.3390/cimb48040403

**Published:** 2026-04-14

**Authors:** Xiaochen Yu, Duopeng An, Dadui Ren, Peng He, Yunkai Yang, Nanye Chen, Rui Wang, Shan Wu, Jun Feng, Meiqing Feng

**Affiliations:** 1 School of Pharmaceutical Sciences, Fudan University, Shanghai 201203, China; yuxc16@fudan.edu.cn (X.Y.); 18111030046@fudan.edu.cn (D.A.); 18111030056@fudan.edu.cn (D.R.); phe20@fudan.edu.cn (P.H.); ykyang17@fudan.edu.cn (Y.Y.); 17211030048@fudan.edu.cn (N.C.); 20211030037@fudan.edu.cn (R.W.); 2 Department of Cell Stress Biology, Roswell Park Comprehensive Cancer Center, Buffalo, NY 14263, USA; shan.wu@roswellpark.org; 3 China State Institute of Pharmaceutical Industry, Shanghai 201203, China

**Keywords:** TRIM24, glucose deprivation, AMPK signaling, aspartate metabolism, metabolic adaptation, nutrient stress

## Abstract

Glucose deprivation is a major metabolic stress that requires coordinated adaptive responses to maintain cellular homeostasis and survival, yet the role of tripartite motif-containing 24 (TRIM24) in this process remains unclear. To address this question, we generated CRISPR-Cas9-mediated TRIM24-knockout MCF-7 and HEK293 cell lines, performed targeted metabolomic profiling and aspartate assays, used 5-aminoimidazole-4-carboxamide-1-β-D-ribofuranoside (AICAR), aminooxyacetic acid (AOA), aspartate supplementation, and glutamic-oxaloacetic transaminase 2 (GOT2) knockdown to probe AMPK signaling and aspartate metabolism, and examined starvation responses in constitutive Trim24 knockout mice on a C57BL/6 background. Loss of TRIM24 sensitized cells to glucose deprivation. Re-expression of TRIM24 partially restored cell viability under glucose deprivation in both MCF-7 and HEK293 cells. Under glucose-free conditions, TRIM24 deficiency was associated with impaired AMP-activated protein kinase (AMPK) pathway activation, increased intracellular aspartate accumulation, and altered ATP/AMP levels. Pharmacological reactivation of AMPK by AICAR improved the survival of TRIM24-deficient cells under glucose deprivation. Reducing intracellular aspartate by AOA treatment or GOT2 knockdown restored AMPK pathway activation and improved adaptation to glucose deprivation, whereas exogenous aspartate suppressed AMPK signaling and increased ATP/AMP levels. In vivo, starvation of Trim24-deficient mice was associated with reduced AMPK pathway activation and increased aspartate levels. Together, these findings support a model in which TRIM24 contributes to adaptation to glucose deprivation and in which abnormal aspartate accumulation contributes to impaired AMPK pathway activation in TRIM24-deficient cells.

## 1. Introduction

Glucose deprivation is a common metabolic stress encountered in diverse physiological and pathological contexts, including fasting, ischemia, and the tumor microenvironment [1]. Because glucose serves as a major source of energy and metabolic intermediates, reduced glucose availability imposes a substantial challenge to cellular homeostasis and survival [2,3,4]. To cope with this stress, cells rapidly adjust their metabolic programs and coordinate adaptive signaling responses that preserve energy balance and sustain viability [5,6]. Failure to mount an appropriate response to glucose limitation results in profound metabolic dysfunction and ultimately cell death [7,8].

AMP-activated protein kinase (AMPK) is a central regulator of the cellular response to energy stress [9]. Under glucose deprivation, AMPK is activated by changes in cellular energy status [10] and promotes adaptive responses that help restore metabolic balance [11,12]. These responses include inhibition of anabolic pathways, modulation of lipid metabolism, and activation of stress-responsive programs through downstream targets such as acetyl-CoA carboxylase (ACC) and Unc-51-like kinase 1 (ULK1) [13]. Proper activation of AMPK is therefore critical for maintaining cell viability during glucose limitation [2].

Tripartite motif-containing 24 (TRIM24), also known as transcription intermediary factor 1α (TIF1α), is a multifunctional regulatory protein implicated in transcriptional control, chromatin regulation, and tumor progression [14]. In addition to its reported E3 ubiquitin ligase activity [15], TRIM24 has also been reported to function as a transcriptional coactivator [16], further highlighting its pleiotropic roles in cellular regulation [17]. Previous studies have shown that TRIM24 participates in cell proliferation [18], differentiation [19], and stress responses [17], and aberrant TRIM24 expression has been linked to multiple human cancers [20]. Despite these observations, the role of TRIM24 in cellular responses to metabolic stress, particularly glucose deprivation, remains incompletely understood.

In addition to canonical signaling pathways, accumulating evidence indicates that metabolic intermediates actively influence stress responses and cell fate decisions. However, whether TRIM24 contributes to adaptation to glucose deprivation through metabolic remodeling remains unclear. In the present study, we show that TRIM24 deficiency impairs cell survival under glucose deprivation and attenuates AMPK pathway activation. Mechanistically, TRIM24 deficiency is associated with abnormal intracellular aspartate accumulation, whereas reducing intracellular aspartate restores AMPK signaling and improves adaptation to glucose deprivation. Together, these findings support a model in which TRIM24 contributes to adaptation to glucose deprivation and in which altered aspartate homeostasis is functionally linked to impaired AMPK pathway activation in TRIM24-deficient cells.

## 2. Materials and Methods

### 2.1. Cell Lines and Cell Culture

MCF-7 and HEK293 cells were obtained from the American Type Culture Collection (ATCC, Manassas, VA, USA). Cells were maintained in Dulbecco’s modified Eagle’s medium (DMEM; Thermo Fisher Scientific, Boston, MA, USA, 11965092) supplemented with 10% fetal bovine serum (FBS; Thermo Fisher Scientific, 10099) and penicillin/streptomycin at 37 °C in a humidified incubator with 5% CO_2_. Cells were routinely tested for mycoplasma contamination.

### 2.2. Generation of TRIM24-Knockout Cells

TRIM24-knockout cell lines were generated using a CRISPR-Cas9 system based on the lentiCRISPR v2 vector. The following human TRIM24 sgRNAs were used:

gRNA1: 5′-CACCGACTCGGGAGGCGGATCCCGT-3′/
       5′-AAACACGGGATCCGCCTCCCGAGTC-3′
gRNA2: 5′-CACCGAACGGCGACGAGCCGCTGAC-3′/
       5′-AAACGTCAGCGGCTCGTCGCCGTTC-3′
gRNA3: 5′-CACCGGAGGCACTAGGTGAACGTAT-3′/
       5′-AAACATACGTTCACCTAGTGCCTCC-3′

Lentiviral transduction was performed using the lentiCRISPR v2 system. After infection, cells were subjected to puromycin selection at 1 μg/mL until uninfected control cells were largely eliminated and the surviving infected cells no longer showed marked ongoing cell death under selection pressure. The selected cell populations were then released from puromycin. For MCF-7 cells, single-cell cloning was subsequently performed by limiting dilution. Two Scramble single-cell clones and eight TRIM24-targeted single-cell-derived clones were retained after initial screening (Figure S1A). Among the TRIM24-targeted clones, Clone1, Clone2, and Clone3 were derived from gRNA1; Clone4, Clone5, and Clone6 were derived from gRNA2; and Clone7 and Clone8 were derived from gRNA3. For HEK293 cells, mixed populations generated using gRNA1, gRNA2, and gRNA3, together with corresponding Scramble control populations, were initially evaluated by immunoblotting (Figure S1B). For MCF-7 cells, the glucose deprivation-sensitive viability phenotype was confirmed in two independent single-cell-derived clones, Clone2 and Clone6, which were derived from gRNA1 and gRNA2, respectively. Based on this independent validation, the detailed mechanistic analyses shown in the main figures, including immunoblotting, aspartate measurement, and related assays, were performed using MCF-7 Clone6 as a representative TRIM24-deficient clone. For HEK293 cells, mixed populations generated using gRNA1, gRNA2, and gRNA3, together with corresponding Scramble control populations, were initially evaluated by immunoblotting. All subsequent HEK293 experiments were performed using the gRNA1-derived mixed population, which showed clear TRIM24 depletion and reproducibly exhibited the glucose deprivation-sensitive phenotype. We chose to continue with a mixed population rather than establishing single-cell-derived HEK293 clones in order to reduce potential clonal variation associated with single-cell isolation.

### 2.3. TRIM24 Re-Expression Experiments

For rescue experiments, puromycin-selected mixed TRIM24-knockout cell populations were first established after lentiviral transduction and maintained in puromycin-containing medium for multiple passages. Before rescue experiments, these cells were cultured for two passages in puromycin-free medium. Cells were then seeded and allowed to attach for 16 h, followed by transient transfection with a pcDNA3.1-Myc-TRIM24 plasmid or the corresponding pcDNA3.1 empty vector using Lipofectamine 2000 (Thermo Fisher Scientific, 11668019) according to the manufacturer’s instructions. Twelve hours after transfection, the medium was replaced with fresh complete growth medium. After an additional 24 h, cells were subjected to glucose deprivation for the indicated times and then analyzed for cell viability. Re-expression of TRIM24 was confirmed by immunoblotting. No additional antibiotic selection or single-cell cloning was performed during the rescue step.

### 2.4. Glucose Deprivation and Other Stress Treatments

For glucose deprivation experiments, cells were cultured in glucose-free DMEM (Thermo Fisher Scientific, 11966025) supplemented with 10% FBS (Thermo Fisher Scientific, 10099). This medium contained glutamine but no sodium pyruvate. For glutamine deprivation, cells were cultured in glutamine-free DMEM (Thermo Fisher Scientific, 11960044) supplemented with 10% FBS under the same conditions. For serum deprivation, cells were cultured in regular high-glucose DMEM without FBS. For hypoxia experiments, cells were maintained in 1% O_2_, 5% CO_2_, and 94% air. Control medium was defined as regular DMEM (Thermo Fisher Scientific, 11965092) supplemented with 10% FBS. Treatment durations are indicated in the corresponding figure legends.

### 2.5. Drug and Metabolite Treatments

5-Aminoimidazole-4-carboxamide-1-β-D-ribofuranoside (AICAR; Selleckchem, Darmstadt, Germany, NSC105823) was used at 500 μM for 6 h. Aminooxyacetic acid (AOA; Selleckchem, S4989) and aspartate (Sigma-Aldrich, Houston, TX, USA, A9978) were used at the concentrations indicated in the corresponding figure legends. For add-back experiments, AOA and aspartate were added simultaneously.

### 2.6. siRNA Transfection

Glutamic-oxaloacetic transaminase 2 (GOT2) knockdown was performed using siRNA purchased from Santa Cruz Biotechnology, Dallas, TX, USA (sc-60052). Cells were transfected with siRNA using Lipofectamine 2000 (Thermo Fisher Scientific) according to the manufacturer’s instructions. Cells were collected 24 h after transfection, and knockdown efficiency was verified by immunoblotting.

### 2.7. Cell Viability and Viable Cell Number Assays

Cell viability was determined by trypan blue exclusion. Cell suspensions were mixed 1:1 with trypan blue (Gibco, Boston, MA, USA, 15250061), incubated for 5 min, and analyzed using an Invitrogen Countess II automated cell counter (Invitrogen, Carlsbad, CA, USA). Cell viability was calculated as the percentage of trypan blue-negative cells relative to total cells. Viable cell number was determined from the number of trypan blue-negative cells and corrected for dilution to estimate the total number of viable cells in each dish.

### 2.8. ATP/AMP Measurement

Intracellular ATP and AMP levels were measured using CellTiter-Glo^TM^ ATP Assay (Promega, Madison, WI, USA, G7570) and AMP-Glo^TM^ Assay (Promega, V5011), respectively, according to the manufacturers’ instructions. Cells cultured under control or glucose-free conditions were collected for analysis. ATP and AMP values were normalized to protein concentration determined by bicinchoninic acid (BCA) assay. ATP/AMP ratios were then calculated and expressed relative to the corresponding control group.

### 2.9. Aspartate Measurement

Intracellular aspartate levels were measured using the Aspartate Assay Kit (Sigma-Aldrich, MAK495) according to the manufacturer’s instructions. For cultured cells, intracellular aspartate was extracted from cell lysates prepared according to the kit protocol. For liver samples, tissue was processed directly according to the kit instructions. Aspartate levels were normalized to protein concentration when applicable and expressed relative to the corresponding control group.

### 2.10. NMR-Based Targeted Metabolomic Analysis

For NMR-based targeted metabolomic analysis, MCF-7 cells were cultured under control conditions or in glucose-free medium for 12 h, then collected, washed three times with PBS, and extracted with ice-cold methanol/water (2:1, *v*/*v*). After three freeze–thaw cycles and repeated extraction, the combined supernatants were lyophilized. The dried extracts were dissolved in phosphate buffer containing 80% D_2_O and 0.001% TSP-d4, centrifuged, and transferred into 5 mm NMR tubes. ^1^H NMR spectra were acquired on a Bruker Avance III 600 MHz spectrometer (Bruker, Billerica, MA, USA) equipped with a Bruker cryoprobe using the standard one-dimensional NOESY pulse sequence for water suppression. NMR analysis was performed at the Analytical Chemistry Platform of the Wuhan Institute of Physics and Mathematics, Chinese Academy of Sciences.

Six independent biological replicates were analyzed per group. Orthogonal partial least squares discriminant analysis (OPLS-DA) was used to evaluate metabolic differences between Scramble and TRIM24-KO cells under each condition. In the OPLS-DA score plots, t[1]P represents the first predictive component, which reflects variation associated with group separation, whereas t[2]O represents the second orthogonal component, which reflects variation unrelated to the group classification. R^2^X indicates the proportion of variation in the metabolite data matrix explained by the model, and Q^2^ indicates the predictive ability of the model estimated by cross-validation.

For visualization of metabolite differences, representative differential ^1^H NMR spectra were displayed alongside the OPLS-DA score plots. In these spectra, the *x*-axis represents chemical shift (ppm), and the *y*-axis represents relative spectral intensity. Resonance peaks at different chemical shifts correspond to protons in distinct chemical environments, and metabolite assignments were made by comparison with reference databases together with spectral interpretation. Within each condition, signals displayed above the baseline indicate metabolites increased in TRIM24-KO cells, whereas signals displayed below the baseline indicate metabolites increased in Scramble cells.

### 2.11. Protein Extraction and Immunoblotting

For protein extraction, culture medium was removed and cells were washed once with PBS. After complete removal of PBS, cells were lysed in radioimmunoprecipitation assay (RIPA) buffer (Beyotime, Shanghai, China, P0013C) supplemented immediately before use with protease inhibitor (Merck, Darmstadt, Germany) and phosphatase inhibitor (Sigma-Aldrich). Lysates were incubated on ice for 1 h, sonicated, and centrifuged at 4 °C for 15 min. Supernatants were collected, and protein concentration was determined using a BCA Protein Assay Kit (Beyotime, P0011). Samples were mixed with denaturing loading buffer, heated at 95 °C for 5 min on a heating block, and subjected to immunoblotting. Membranes were blocked with 5% non-fat milk and incubated with primary antibodies overnight at 4 °C. After washing, membranes were incubated with horseradish peroxidase (HRP)-conjugated secondary antibodies for 1 h at room temperature. All antibodies were diluted in 5% non-fat milk. Proteins were detected using an enhanced chemiluminescence substrate.

The following primary antibodies were used: TRIM24 (Proteintech, Wuhan, China, 14208-1-AP; 1:2000), phospho-AMPK(T172) (Cell Signaling Technology, Boston, MA, USA, 2535; 1:1000), AMPK (Cell Signaling Technology, 2795; 1:1000), phospho-ACC(S79) (Cell Signaling Technology, 11818; 1:1000), ACC (Cell Signaling Technology, 3676; 1:1000), phospho-ULK1(S555) (Cell Signaling Technology, 5869; 1:1000), ULK1 (Cell Signaling Technology, 8054; 1:1000), GOT2 (Santa Cruz Biotechnology, sc-271702; 1:1000), GAPDH (Cell Signaling Technology, 2118; 1:3000), β-actin (Cell Signaling Technology, 4967; 1:3000), and Tubulin (Cell Signaling Technology, 2146; 1:3000). HRP-conjugated secondary antibodies against rabbit IgG (Cell Signaling Technology, 7074; 1:5000) and mouse IgG (Cell Signaling Technology, 7076; 1:5000) were used.

### 2.12. Densitometric Analysis

Immunoblot band intensities were quantified using ImageJ software V10.2. For signaling readouts, phospho-protein signals were normalized to the corresponding total protein signals, and values were then expressed relative to the first control lane, which was set to 1.0. In experiments in which total protein signals were not suitable for reliable quantification, phospho-protein signals were quantified alone and normalized to the first control lane.

### 2.13. Mouse Model

A constitutive Trim24 knockout mouse line on a C57BL/6 background was generated using a CRISPR-Cas9 strategy targeting exon 2 of the mouse Trim24 gene. An 1854-bp fragment within exon 2 was deleted, resulting in the complete loss of TRIM24 expression. Genome-edited sperm cells carrying the Trim24 deletion were introduced into oocytes by micromanipulation, embryos were transferred into recipient females, and founder mice were subsequently expanded by breeding to establish a stable colony. After genotyping, homozygous Trim24 knockout mice and littermate controls were used for experiments. Male mice at 7–8 weeks of age were used. All animal procedures were approved by the Institutional Animal Care and Use Committee (IACUC) of Fudan University (IACUC approval no. 2022-03-SY-FMQ-50).

### 2.14. Mouse Genotyping

Mouse tail genomic DNA was used for genotyping. The following primers were used:

F1: 5′-CCACTAAGCCCAGTTCCCGAG-3′
F2: 5′-TGTCTCTCATCCCCAGGGCTTTAC-3′
R1: 5′-GCCCTAAATCCCACTGGTACAAAC-3′

Two PCR strategies were used. In strategy 1, primers F1 and R1 flank the deleted 1854-bp region within exon 2. In principle, the WT allele yields a 2456-bp product, whereas the deleted allele yields a 596-bp product. Under the PCR conditions used here, the 2456-bp WT fragment was not efficiently amplified, whereas the deleted allele produced a detectable band at approximately 596 bp. Therefore, WT mice were negative in this reaction, whereas heterozygous and homozygous knockout mice were positive.

In strategy 2, primers F2 and R1 amplify a 346-bp fragment within the intact exon 2 region. WT and heterozygous mice yielded the 346-bp product, whereas homozygous knockout mice showed no amplification because the F2 binding region was deleted. Thus, combined interpretation of both PCR reactions allowed identification of all genotypes: WT mice were negative in strategy 1 and positive in strategy 2; heterozygous mice were positive in both strategies; homozygous knockout mice were positive in strategy 1 and negative in strategy 2.

### 2.15. Starvation Experiment in Mice

For starvation experiments, male mice were deprived of food for 48 h with free access to water. Body weight was monitored during the starvation period. After 48 h, liver and blood samples were collected. Liver samples used for immunoblotting were rapidly frozen in liquid nitrogen and processed according to the protein extraction procedure described above. Liver samples used for aspartate measurement were processed directly according to the aspartate assay protocol.

### 2.16. Statistical Analysis

Data are presented as mean ± SD. The value of *n* represents independent biological replicates unless otherwise indicated. Statistical analyses were performed using GraphPad Prism 8. Student’s *t*-test, one-way ANOVA followed by Tukey’s multiple-comparisons test, or two-way ANOVA was applied as appropriate. A value of *p* < 0.05 was considered statistically significant.

## 3. Results

### 3.1. Loss of TRIM24 Sensitizes Cells to Glucose Deprivation

To investigate the role of TRIM24 in metabolic stress responses, we generated TRIM24-deficient MCF-7 and HEK293 models using a CRISPR-Cas9 system based on lentiCRISPR v2. In MCF-7 cells, two Scramble single-cell clones and eight TRIM24-targeted single-cell-derived clones were analyzed by immunoblotting (Figure S1A). Among the TRIM24-targeted clones, Clone1, Clone2, and Clone3 were derived from gRNA1; Clone4, Clone5, and Clone6 were derived from gRNA2; and Clone7 and Clone8 were derived from gRNA3. In HEK293 cells, mixed populations generated using gRNA1, gRNA2, and gRNA3, together with corresponding Scramble control populations, were initially evaluated by immunoblotting, and all subsequent HEK293 experiments were performed using the gRNA1-derived mixed population (Figure S1B).

**Figure 1:**
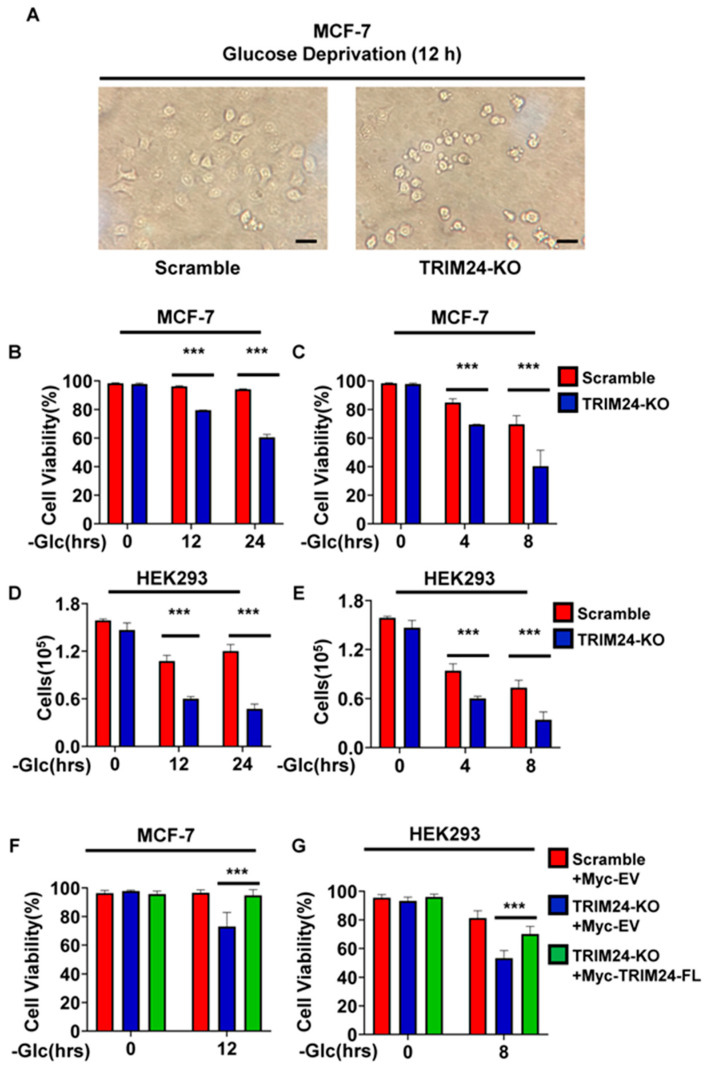
Loss of TRIM24 sensitizes MCF-7 and HEK293 cells to glucose deprivation. (**A**) Representative bright-field images of Scramble and TRIM24-KO MCF-7 cells cultured in glucose-free medium for 12 h. (**B**) Cell viability of Scramble and TRIM24-KO MCF-7 cells cultured in glucose-free medium for the indicated times (0, 12, and 24 h). Cell viability was determined by trypan blue staining and calculated as the percentage of viable cells relative to total cells. (**C**) Cell viability of Scramble and TRIM24-KO HEK293 cells cultured in glucose-free medium for the indicated times (0, 4, and 8 h). Cell viability was determined by trypan blue staining and calculated as the percentage of viable cells relative to total cells. (**D**) Viable cell numbers of Scramble and TRIM24-KO MCF-7 cells cultured in glucose-free medium for the indicated times. Cell numbers were determined by trypan blue staining followed by counting of all viable cells. (**E**) Viable cell numbers of Scramble and TRIM24-KO HEK293 cells cultured in glucose-free medium for the indicated times. Cell numbers were determined by trypan blue staining followed by counting of all viable cells. (**F**) Cell viability of Scramble + empty vector, TRIM24-KO + empty vector, and TRIM24-KO + Myc-TRIM24 full-length MCF-7 cells cultured in glucose-free medium for the indicated times (0 and 12 h). Rescue was performed by transient transfection in established TRIM24-KO mixed populations. Re-expression of TRIM24 was confirmed by immunoblotting in Figure S1C. (**G**) Cell viability of Scramble + empty vector, TRIM24-KO + empty vector, and TRIM24-KO + Myc-TRIM24 full-length HEK293 cells cultured in glucose-free medium for the indicated times (0 and 8). Rescue was performed by transient transfection in established TRIM24-KO mixed populations. Re-expression of TRIM24 was confirmed by immunoblotting in Figure S1D. Data are presented as mean ± SD from three independent experiments (*n* = 3). *** *p* < 0.001.

We first examined whether TRIM24 deficiency affects cellular responses to glucose deprivation. Under glucose-free conditions, TRIM24-deficient MCF-7 cells exhibited more pronounced morphological changes, including cell rounding and detachment, than Scramble control cells (Figure 1A). Consistent with these observations, loss of TRIM24 significantly reduced cell viability and viable cell number under glucose deprivation in both MCF-7 and HEK293 cells (Figure 1B–E). In MCF-7 cells, this glucose deprivation-sensitive viability phenotype was confirmed in two independent single-cell-derived clones, Clone2 and Clone6, which were generated using different sgRNAs. On this basis, subsequent mechanistic analyses in MCF-7 cells were performed using Clone6 as a representative TRIM24-targeted clone, in order to avoid redundancy while retaining a model in which the phenotype had already been independently validated. In HEK293 cells, all subsequent experiments were performed using the gRNA1-derived mixed population, which showed clear TRIM24 depletion and reproducibly exhibited the glucose deprivation-sensitive phenotype. This mixed population was used for subsequent analyses in part to reduce potential clonal variation associated with single-cell isolation. These results indicate that TRIM24 supports efficient adaptation to glucose deprivation.

To determine whether this phenotype was attributable to loss of TRIM24 rather than clonal variation, we next performed re-expression experiments in TRIM24-deficient cells. For these rescue assays, established TRIM24-KO mixed populations were first maintained under puromycin selection for multiple passages after lentiviral transduction, then cultured for two passages in puromycin-free medium before transient transfection with pcDNA3.1-based constructs. Re-expression of Myc-TRIM24 in TRIM24-KO MCF-7 and HEK293 cells was confirmed by immunoblotting (Figure S1C,D). Under glucose-free conditions, re-expression of TRIM24 partially restored cell viability in both MCF-7 and HEK293 cells compared with empty vector-transfected TRIM24-KO cells (Figure 1F,G). These findings further support the conclusion that the glucose deprivation-sensitive phenotype is attributable to loss of TRIM24.

We next asked whether this phenotype reflects a broad defect in multiple stress responses or is more prominent under glucose deprivation. To address this question, we examined the effects of glutamine deprivation, serum deprivation, and hypoxia in MCF-7 cells. In contrast to the marked phenotype observed under glucose-free conditions, loss of TRIM24 did not significantly alter cell viability or viable cell number under glutamine deprivation, serum deprivation, or hypoxic conditions (Figure S1E–J). These findings suggest that, among the stress conditions tested here, the effect of TRIM24 loss was most pronounced under glucose deprivation.

Given the prominent survival phenotype observed under glucose deprivation and the central role of AMPK in the response to energy stress [21], we next examined ATP/AMP ratio dynamics during glucose deprivation. In both MCF-7 and HEK293 cells, the ATP/AMP ratio declined over time after glucose withdrawal. However, the decrease was more rapid and more pronounced in control cells, whereas TRIM24-deficient cells showed a less pronounced decline, resulting in a relatively higher ATP/AMP ratio at matched time points (Figure S1K,L). Together, these results suggest that TRIM24 deficiency alters the temporal energetic response to glucose deprivation and prompted us to further examine whether TRIM24 regulates AMPK pathway activation during glucose deprivation.

### 3.2. Loss of TRIM24 Impairs AMPK Pathway Activation Under Glucose Deprivation

**Figure 2:**
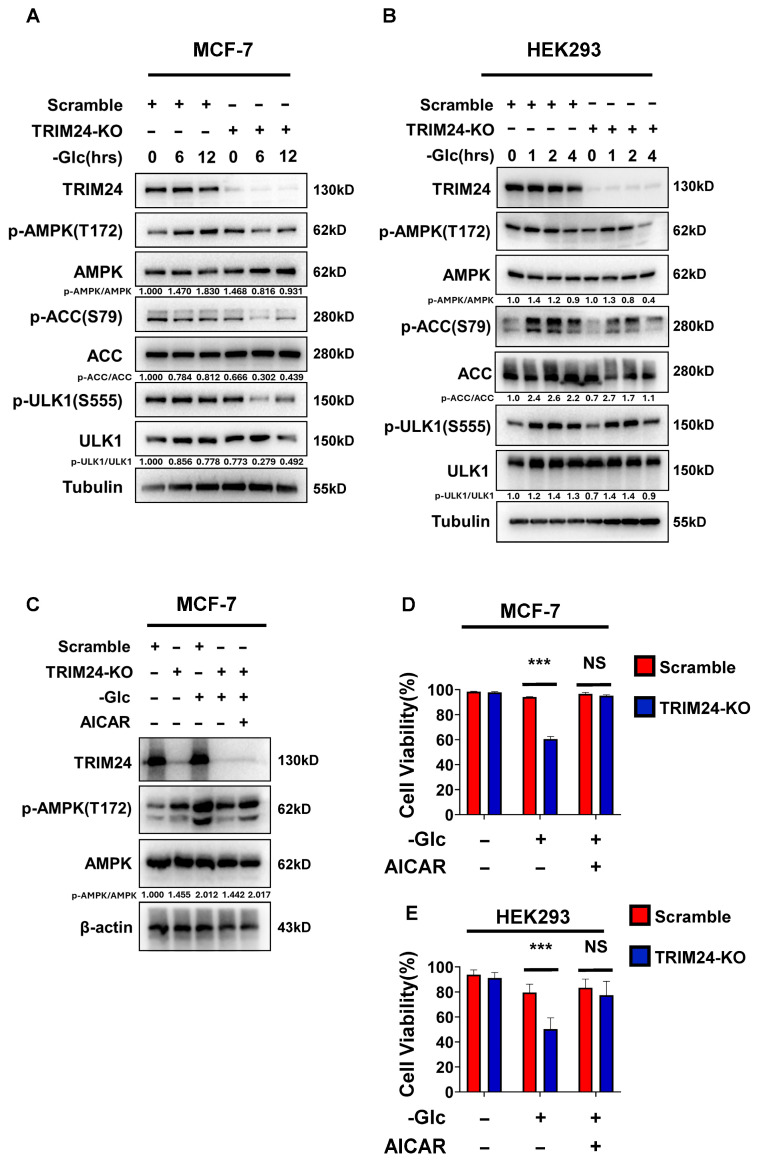
Loss of TRIM24 impairs AMPK pathway activation under glucose deprivation. (**A**) Immunoblot analysis of TRIM24, phospho-AMPK (Thr172), total AMPK, phospho-ACC (Ser79), total ACC, phospho-ULK1 (Ser555), total ULK1, and tubulin in Scramble and TRIM24-KO MCF-7 cells cultured in glucose-free medium for the indicated times (0, 6, and 12 h). Tubulin was used as a loading control. Densitometric values of p-AMPK/AMPK, p-ACC/ACC, and p-ULK1/ULK1 are indicated below the blots. (**B**) Immunoblot analysis of TRIM24, phospho-AMPK (Thr172), total AMPK, phospho-ACC (Ser79), total ACC, phospho-ULK1 (Ser555), total ULK1, and tubulin in Scramble and TRIM24-KO HEK293 cells cultured in glucose-free medium for the indicated times (0, 1, 2, and 4 h). Tubulin was used as a loading control. Densitometric values of p-AMPK/AMPK, p-ACC/ACC, and p-ULK1/ULK1 are indicated below the blots. (**C**) Immunoblot analysis of TRIM24, phospho-AMPK (Thr172), total AMPK, and β-actin in Scramble and TRIM24-KO MCF-7 cells cultured under control conditions, in glucose-free medium alone, or in glucose-free medium supplemented with AICAR. β-actin was used as a loading control. Densitometric values of p-AMPK/AMPK are indicated below the blots. (**D**) Cell viability of Scramble and TRIM24-KO MCF-7 cells cultured under control conditions, in glucose-free medium alone, or in glucose-free medium supplemented with AICAR. Cell viability was determined by trypan blue staining and calculated as the percentage of viable cells relative to total cells. (**E**) Cell viability of Scramble and TRIM24-KO HEK293 cells cultured under control conditions, in glucose-free medium alone, or in glucose-free medium supplemented with AICAR. Cell viability was determined by trypan blue staining and calculated as the percentage of viable cells relative to total cells. Data are presented as mean ± SD from three independent experiments (*n* = 3). NS, not significant; *** *p* < 0.001.

Given the phenotype under glucose deprivation, we next asked whether TRIM24 affects activation of the AMPK pathway under this condition. In MCF-7 cells, glucose deprivation induced phosphorylation of AMPK at Thr172 in Scramble cells, whereas this response was attenuated in TRIM24-deficient cells (Figure 2A). Consistent with reduced AMPK activation, phosphorylation of the downstream AMPK targets ACC at Ser79 and ULK1 at Ser555 was also decreased in TRIM24-deficient cells. Densitometric analysis of p-AMPK/AMPK, p-ACC/ACC, and p-ULK1/ULK1 supported impaired AMPK pathway activation in TRIM24-deficient cells under glucose-free conditions. A similar pattern was observed in HEK293 cells, in which glucose deprivation induced phosphorylation of AMPK, ACC, and ULK1 in Scramble cells but to a lesser extent in the TRIM24-deficient mixed population (Figure 2B). These results suggest that TRIM24 contributes to efficient activation of the AMPK pathway under glucose deprivation.

To determine whether impaired AMPK activation contributes functionally to the reduced survival of TRIM24-deficient cells, we next examined the effect of pharmacological AMPK activation. AICAR, a pharmacological activator of AMPK, restored AMPK phosphorylation in TRIM24-deficient MCF-7 cells under glucose deprivation (Figure 2C). Importantly, AICAR also improved the viability of TRIM24-deficient cells under glucose-free conditions in both MCF-7 and HEK293 cells, attenuating the difference between Scramble and TRIM24-deficient cells (Figure 2D,E). Together, these findings suggest that defective AMPK activation is an important contributor to the glucose deprivation-sensitive phenotype associated with TRIM24 deficiency.

### 3.3. Loss of TRIM24 Is Associated with Increased Aspartate Accumulation Under Glucose Deprivation

Because TRIM24-deficient cells displayed both impaired AMPK activation and higher ATP/AMP levels under glucose deprivation, we next asked whether loss of TRIM24 is associated with altered intracellular metabolite accumulation during glucose deprivation. During routine observation of glucose-deprived cultures, we noted an apparent difference in medium color between Scramble and TRIM24-deficient cells, which prompted us to further examine metabolic alterations by NMR-based targeted metabolomic analysis.

NMR-based metabolomic analysis was performed in MCF-7 cells cultured under control conditions or glucose deprivation for 12 h. OPLS-DA score plots indicated that the metabolic profiles of Scramble and TRIM24-deficient cells differed under both conditions, with more evident separation under glucose deprivation (Figure S2). The corresponding differential ^1^H NMR spectra further showed that the metabolic alterations associated with TRIM24 deficiency differed between normal medium and glucose-deprived conditions. Within each condition, metabolites elevated in TRIM24-deficient cells and those elevated in Scramble cells were displayed separately for clarity. Notably, aspartate was elevated in TRIM24-deficient cells under both conditions, but the increase was more prominent under glucose deprivation than under control conditions.

**Figure 3:**
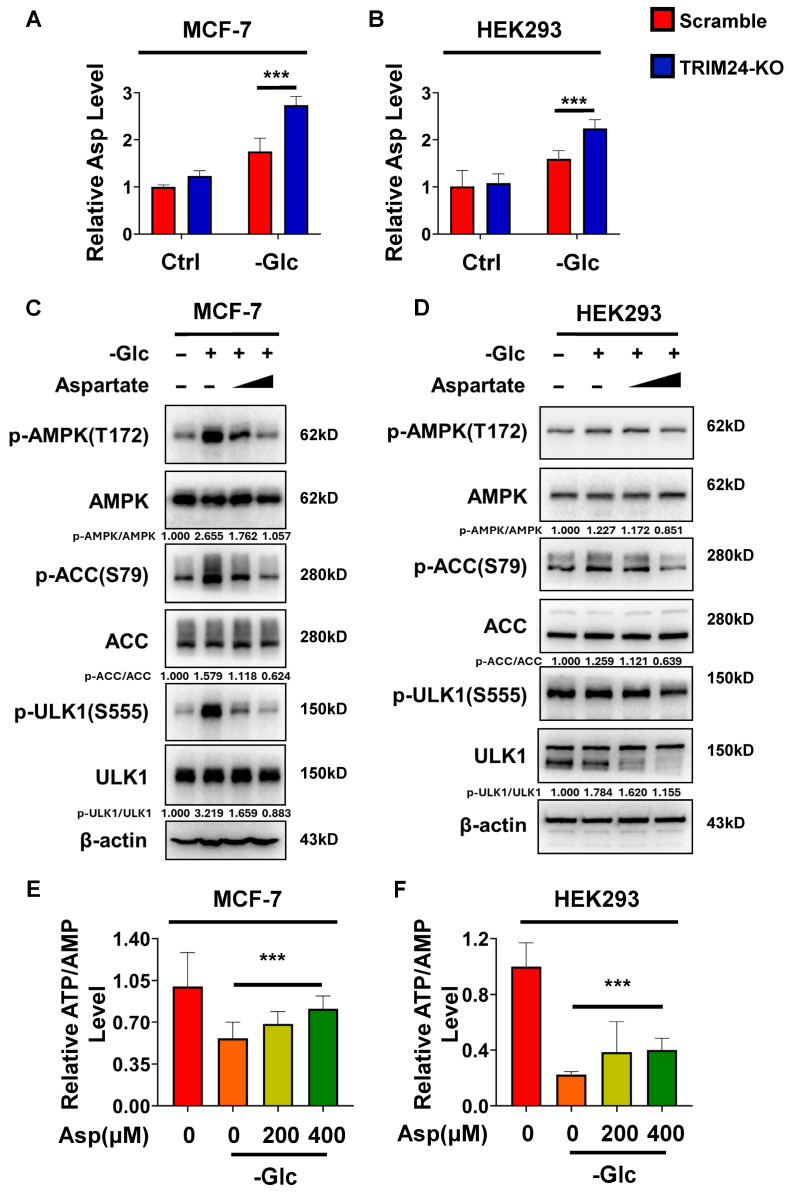
Loss of TRIM24 is associated with increased aspartate accumulation and altered AMPK pathway activation under glucose deprivation. (**A**) Relative aspartate levels in Scramble and TRIM24-KO MCF-7 cells cultured under control conditions or in glucose-free medium, as determined using an aspartate assay kit. (**B**) Relative aspartate levels in Scramble and TRIM24-KO HEK293 cells cultured under control conditions or in glucose-free medium, as determined using an aspartate assay kit. (**C**) Immunoblot analysis of phospho-AMPK (Thr172), total AMPK, phospho-ACC (Ser79), total ACC, phospho-ULK1 (Ser555), total ULK1, and β-actin in MCF-7 cells cultured under control conditions, in glucose-free medium alone, or in glucose-free medium supplemented with aspartate (200 or 400 μM). β-actin was used as a loading control. Densitometric values of p-AMPK/AMPK, p-ACC/ACC, and p-ULK1/ULK1 are indicated below the blots. (**D**) Immunoblot analysis of phospho-AMPK (Thr172), total AMPK, phospho-ACC (Ser79), total ACC, phospho-ULK1 (Ser555), total ULK1, and β-actin in HEK293 cells cultured under control conditions, in glucose-free medium alone, or in glucose-free medium supplemented with aspartate (200 or 400 μM). β-actin was used as a loading control. Densitometric values of p-AMPK/AMPK, p-ACC/ACC, and p-ULK1/ULK1 are indicated below the blots. (**E**) Relative ATP/AMP levels in MCF-7 cells cultured under control conditions, in glucose-free medium alone, or in glucose-free medium supplemented with aspartate (200 or 400 μM). (**F**) Relative ATP/AMP levels in HEK293 cells cultured under control conditions, in glucose-free medium alone, or in glucose-free medium supplemented with aspartate (200 or 400 μM). Data are presented as mean ± SD from three independent experiments (*n* = 3). *** *p* < 0.001.

To independently validate this finding, we measured intracellular aspartate levels using an aspartate assay kit. Consistent with the metabolomic analysis, TRIM24-deficient cells exhibited significantly higher aspartate levels than Scramble cells under glucose-free conditions in both MCF-7 and HEK293 cells, whereas the difference was much less apparent under control conditions (Figure 3A,B).

A previous study by Zhu et al. showed that aspartate sustains ATP/AMP levels during starvation, attenuates AMPK activation, and impairs adaptation to nutrient stress [22]. We therefore asked whether elevated aspartate might contribute to the altered response to glucose deprivation associated with TRIM24 deficiency. To test this possibility, we supplemented cells with exogenous aspartate under glucose-free conditions and examined AMPK pathway activation. In MCF-7 cells, aspartate supplementation reduced phosphorylation of AMPK, ACC, and ULK1 in a dose-dependent manner (Figure 3C). Similar effects were observed in HEK293 cells, in which exogenous aspartate also attenuated phosphorylation of AMPK, ACC, and ULK1 under glucose deprivation (Figure 3D). Densitometric analysis further supported suppression of AMPK pathway activation by aspartate in both cell lines.

We next examined whether aspartate also affects cellular energy status in our model. Under glucose-free conditions, aspartate supplementation increased ATP/AMP levels in both MCF-7 and HEK293 cells (Figure 3E,F). Thus, elevated aspartate was sufficient to attenuate AMPK pathway activation and was associated with higher ATP/AMP levels during glucose deprivation. Together, these findings support the idea that abnormal aspartate accumulation contributes to impaired AMPK activation under glucose deprivation associated with TRIM24 deficiency.

### 3.4. Aspartate Modulation Alters Glucose Deprivation Responses in TRIM24-Deficient Cells

**Figure 4:**
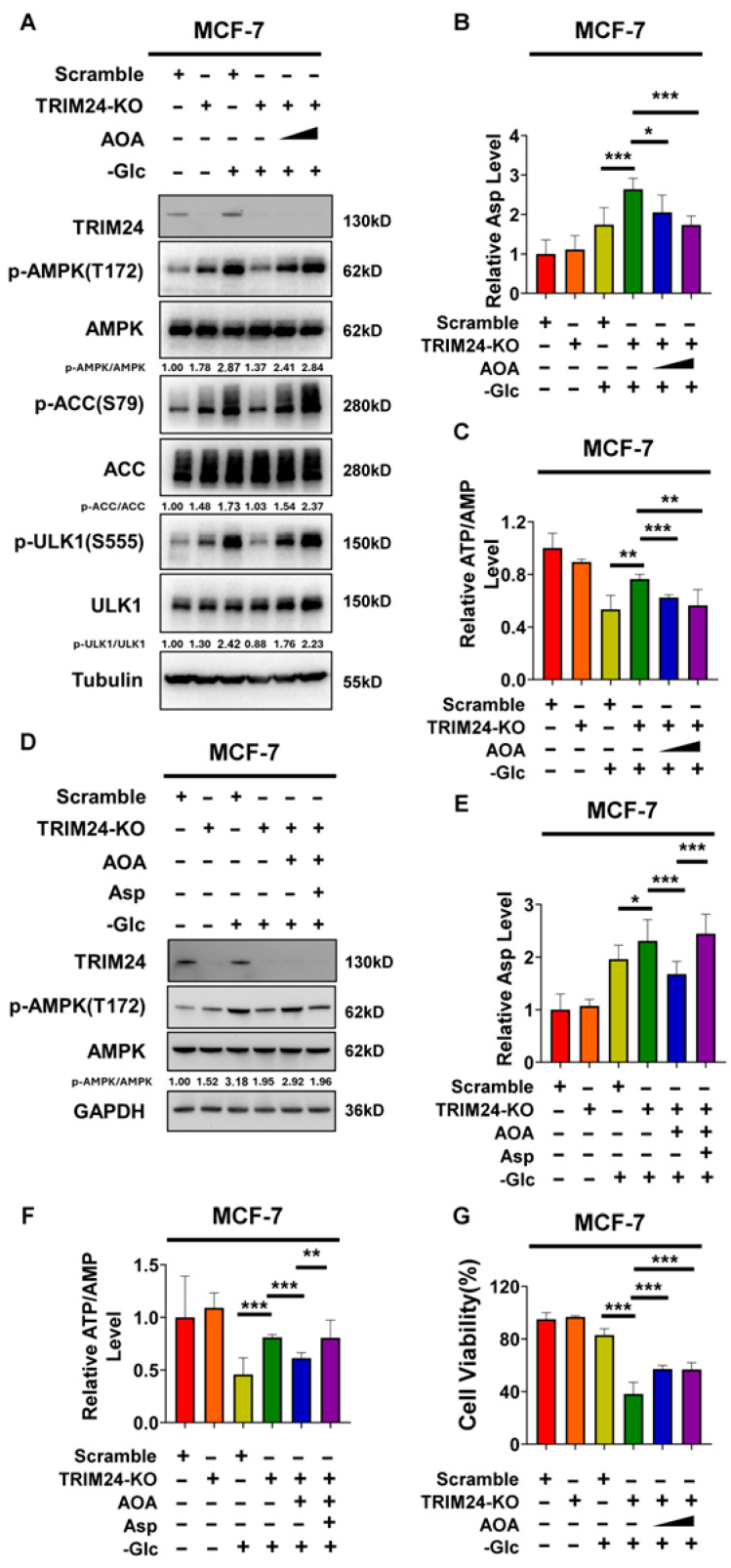
Aspartate modulation alters glucose deprivation responses in TRIM24-deficient MCF-7 cells. (**A**) Immunoblot analysis of TRIM24, phospho-AMPK (Thr172), total AMPK, phospho-ACC (Ser79), total ACC, phospho-ULK1 (Ser555), total ULK1, and tubulin in Scramble and TRIM24-KO MCF-7 cells cultured under control conditions, in glucose-free medium alone, or in glucose-free medium supplemented with aminooxyacetic acid (AOA; 500 μM or 1 mM). Tubulin was used as a loading control. Densitometric values of p-AMPK/AMPK, p-ACC/ACC, and p-ULK1/ULK1 are indicated below the blots. (**B**) Intracellular aspartate levels in Scramble and TRIM24-KO MCF-7 cells cultured under control conditions or in glucose-free medium in the absence or presence of AOA (500 μM or 1 mM), as determined using an aspartate assay kit. (**C**) Relative ATP/AMP levels in Scramble and TRIM24-KO MCF-7 cells cultured under control conditions or in glucose-free medium in the absence or presence of AOA (500 μM or 1 mM). (**D**) Immunoblot analysis of TRIM24, phospho-AMPK (Thr172), total AMPK, and GAPDH in Scramble and TRIM24-KO MCF-7 cells cultured under control conditions, in glucose-free medium alone, in glucose-free medium supplemented with AOA (500 μM), or in glucose-free medium supplemented with AOA (500 μM) and aspartate (400 μM). GAPDH was used as a loading control. Densitometric values of p-AMPK/AMPK are indicated below the blots. (**E**) Intracellular aspartate levels in Scramble and TRIM24-KO MCF-7 cells cultured under control conditions, in glucose-free medium alone, in glucose-free medium supplemented with AOA (500 μM), or in glucose-free medium supplemented with AOA (500 μM) and aspartate (400 μM), as determined using an aspartate assay kit. (**F**) Relative ATP/AMP levels in Scramble and TRIM24-KO MCF-7 cells cultured under control conditions, in glucose-free medium alone, in glucose-free medium supplemented with AOA (500 μM), or in glucose-free medium supplemented with AOA (500 μM) and aspartate (400 μM). (**G**) Cell viability of Scramble and TRIM24-KO MCF-7 cells cultured under control conditions or in glucose-free medium in the absence or presence of AOA (500 μM or 1 mM). Cell viability was determined by trypan blue staining and calculated as the percentage of viable cells relative to total cells. Data are presented as mean ± SD from three independent experiments (*n* = 3). * *p* < 0.05; ** *p* < 0.01; *** *p* < 0.001.

To determine whether elevated aspartate is functionally involved in the defective response to glucose deprivation associated with TRIM24 deficiency, we first asked whether decreasing intracellular aspartate could rescue this phenotype. We next used aminooxyacetic acid (AOA), a transaminase inhibitor reported to inhibit glutamic-oxaloacetic transaminase 2 (GOT2) [23]. In MCF-7 cells, treatment with AOA under glucose-free conditions increased phosphorylation of AMPK, ACC, and ULK1 in TRIM24-deficient cells, and this effect was partially dose-dependent (Figure 4A). Consistent with these signaling changes, intracellular aspartate levels measured by an aspartate assay kit were significantly reduced by AOA treatment in TRIM24-deficient cells under glucose deprivation (Figure 4B). AOA treatment also lowered ATP/AMP levels in TRIM24-deficient cells toward those observed in Scramble cells (Figure 4C). These findings support the idea that reducing intracellular aspartate helps restore AMPK pathway activation and partially normalize the altered energetic state associated with TRIM24 deficiency during glucose deprivation.

To further test whether these rescue effects were mediated, at least in part, through aspartate, we selected one concentration of AOA and performed aspartate add-back experiments. Under glucose-free conditions, AOA increased AMPK phosphorylation in TRIM24-deficient MCF-7 cells, whereas re-addition of aspartate largely reversed this effect (Figure 4D). Measurement of intracellular aspartate confirmed that AOA reduced aspartate accumulation in TRIM24-deficient cells, and that exogenous aspartate restored intracellular aspartate levels (Figure 4E). In parallel, the reduction in ATP/AMP levels induced by AOA was also reversed by aspartate re-addition (Figure 4F). Together, these data support the conclusion that the effects of AOA on AMPK signaling and ATP/AMP status are mediated, at least in part, through limiting intracellular aspartate accumulation.

We next examined whether reducing intracellular aspartate also affects cell survival under glucose deprivation. AOA significantly improved the viability of TRIM24-deficient MCF-7 cells under glucose-free conditions, supporting the idea that abnormal aspartate accumulation contributes to the defective adaptation of TRIM24-deficient cells to glucose deprivation.

To determine whether this mechanism is also operative in another cell type, we performed parallel experiments in HEK293 cells. Similar to the results obtained in MCF-7 cells, AOA treatment increased AMPK phosphorylation in the TRIM24-deficient HEK293 mixed population under glucose deprivation, and this effect was reversed by aspartate re-addition (Figure S3A). AOA also reduced intracellular aspartate levels and decreased ATP/AMP levels, whereas exogenous aspartate restored both readouts (Figure S3B,C). In addition, AOA improved the viability of the TRIM24-deficient HEK293 mixed population under glucose-free conditions, and aspartate re-addition partially attenuated this effect (Figure S3F). A similar trend was also observed in MCF-7 cells (Figure S3G).

As an independent approach to decrease intracellular aspartate, we next targeted GOT2 in HEK293 cells. Among the three siRNAs tested, one achieved the strongest knockdown efficiency, whereas the other two also effectively reduced GOT2 expression (Figure S3D). GOT2 knockdown increased AMPK phosphorylation in the TRIM24-deficient HEK293 mixed population under glucose deprivation, and this effect was again reduced by exogenous aspartate (Figure S3E). Together, these findings from both pharmacological and genetic approaches further support the idea that elevated intracellular aspartate is a functionally relevant mediator linking TRIM24 deficiency to impaired AMPK activation and defective adaptation to glucose deprivation.

### 3.5. TRIM24 Deficiency Alters Starvation Responses In Vivo

To further examine the physiological relevance of our findings, we generated a constitutive Trim24 knockout mouse model on a C57BL/6 background. In this model, a genomic fragment within exon 2 of the mouse Trim24 gene was deleted by CRISPR-Cas9, resulting in complete loss of TRIM24 expression. Genotyping was performed using a dual-PCR strategy (Figure S4A). The F1/R1 primer pair detected the deleted Trim24 allele, whereas the F2/R1 primer pair detected the intact WT allele. Accordingly, WT mice were identified by the presence of the lower F2/R1 PCR product alone, heterozygous mice by the presence of both PCR products, and homozygous Trim24 knockout mice by the presence of the upper F1/R1 PCR product together with the absence of the lower F2/R1 PCR product.

We next monitored body weight changes during starvation. At baseline, Trim24^−/−^ mice showed a lower body weight than age-matched Trim24^+/+^ mice. Starvation led to progressive body weight loss in both groups, and Trim24^−/−^ mice showed a trend toward more rapid weight reduction over time, although this difference did not reach statistical significance under the conditions tested (Figure S4B).

**Figure 5:**
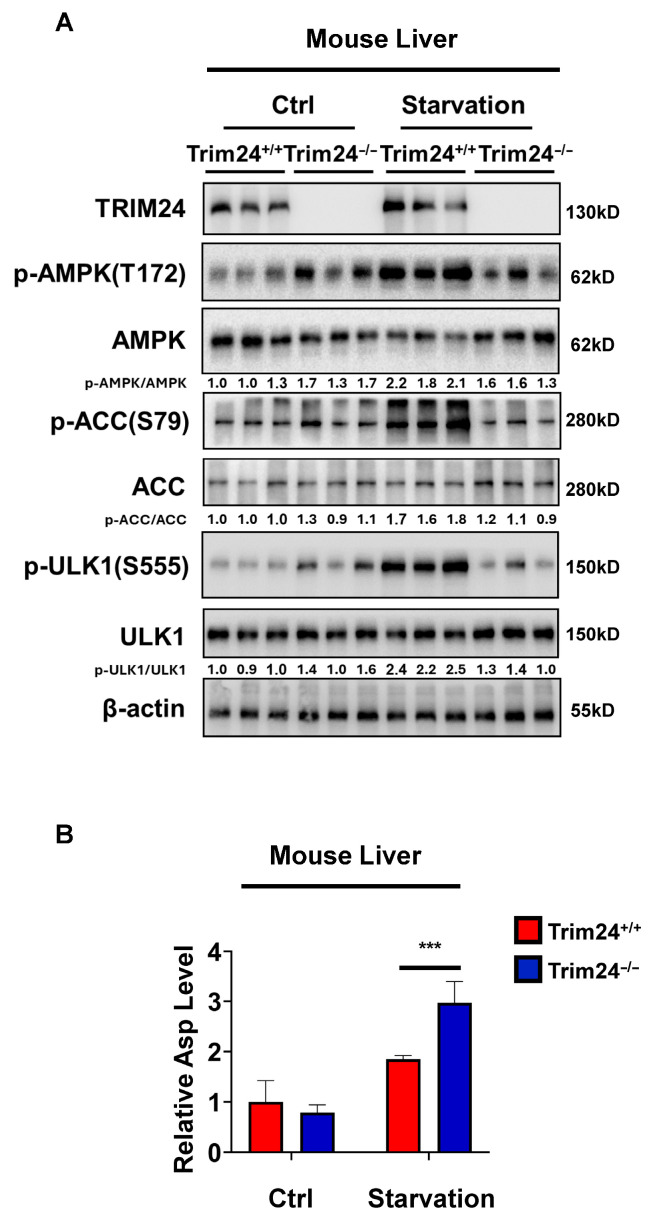
TRIM24 deficiency alters starvation responses in vivo. (**A**) Immunoblot analysis of TRIM24, phospho-AMPK (Thr172), total AMPK, phospho-ACC (Ser79), total ACC, phospho-ULK1 (Ser555), total ULK1, and β-actin in samples collected from Trim24^+/+^ and Trim24^−/−^ mice under control conditions or after 48 h starvation. β-actin was used as a loading control. Densitometric values of p-AMPK/AMPK, p-ACC/ACC, and p-ULK1/ULK1 are indicated below the blots. (**B**) Relative aspartate levels in samples collected from Trim24^+/+^ and Trim24^−/−^ mice under control conditions or after 48 h starvation. Data are presented as mean ± SD from three independent experiments (*n* = 3). *** *p* < 0.001.

We then examined AMPK pathway activation under starvation conditions in vivo. In samples collected from control mice, basal phosphorylation of AMPK, ACC, and ULK1 was low in both genotypes (Figure 5A). Starvation for 48 h induced phosphorylation of AMPK at Thr172, ACC at Ser79, and ULK1 at Ser555 in Trim24^+/+^ mice, whereas this response was attenuated in Trim24^−/−^ mice. Densitometric analysis of p-AMPK/AMPK, p-ACC/ACC, and p-ULK1/ULK1 further supported reduced activation of the AMPK pathway in Trim24-deficient mice under starvation conditions (Figure 5A).

Because our in vitro studies also showed that TRIM24 deficiency was associated with abnormal aspartate accumulation under glucose deprivation, we next measured aspartate levels in vivo. Under control conditions, aspartate levels were comparable between Trim24^+/+^ and Trim24^−/−^ mice. In contrast, starvation was associated with increased aspartate levels in Trim24^−/−^ mice compared with Trim24^+/+^ mice (Figure 5B). Together, these findings provide in vivo evidence consistent with the cell-based observations.

## 4. Discussion

In this study, we provide evidence that TRIM24 contributes to adaptation to glucose deprivation. Loss of TRIM24 sensitized MCF-7 and HEK293 cells to glucose deprivation, while having little effect under glutamine deprivation, serum deprivation, or hypoxic conditions in the settings tested here. Mechanistically, TRIM24 deficiency was associated with impaired AMPK pathway activation, increased intracellular aspartate accumulation, and altered ATP/AMP levels under glucose-free conditions. Exogenous aspartate suppressed AMPK signaling and increased ATP/AMP levels, whereas reducing intracellular aspartate by AOA treatment or GOT2 knockdown restored AMPK pathway activation and improved cellular adaptation to glucose deprivation. In addition, starvation experiments in Trim24-deficient mice showed reduced AMPK pathway activation together with increased aspartate levels in vivo. Importantly, re-expression of TRIM24 partially restored cell viability under glucose deprivation in both MCF-7 and HEK293 cells, further supporting the conclusion that the phenotype is attributable to loss of TRIM24. Collectively, these findings support a model in which TRIM24 contributes to adaptation to glucose deprivation and in which altered aspartate homeostasis is functionally linked to impaired AMPK signaling in TRIM24-deficient cells.

A notable feature of our results is that the phenotype was most pronounced under glucose deprivation among the stress conditions examined in this study. Although TRIM24 has been implicated in a broad range of cellular processes, its loss did not produce comparable defects under glutamine deprivation, serum deprivation, or hypoxia in our experimental setting. This suggests that TRIM24 is not broadly required for all stress responses, but instead may play a more prominent role during adaptation to glucose limitation. Our data suggest that TRIM24 acts upstream of AMPK pathway activation under glucose deprivation, a central component of the cellular response to energy stress. In TRIM24-deficient cells, glucose deprivation failed to induce AMPK, ACC, and ULK1 phosphorylation to the same extent as in control cells, and pharmacological activation of AMPK by AICAR alleviated the glucose deprivation-sensitive phenotype. These observations suggest that defective AMPK activation is an important contributor to the impaired survival of TRIM24-deficient cells under glucose limitation. Notably, under nutrient-replete conditions, TRIM24 deficiency was associated with a modest increase in AMPK phosphorylation, a pattern that was reproducibly observed in our experiments and is consistent with a previous report [24], suggesting that the effect of TRIM24 on AMPK signaling may depend on nutrient context. Together with our glucose deprivation data, this suggests that TRIM24 may influence AMPK signaling in a context-dependent manner, with distinct effects under nutrient-replete and nutrient-stressed conditions. Recent work has also shown that TRIM24 can promote cellular adaptation to energy stress through regulation of ULK1, further supporting the idea that TRIM24 contributes to stress adaptation under metabolically challenging conditions [25].

Our data further support a role for aspartate as a functionally relevant metabolic mediator linking TRIM24 deficiency to impaired AMPK signaling during glucose deprivation. NMR-based metabolomic analysis revealed altered metabolic profiles in TRIM24-deficient cells under both control and glucose-deprived conditions, with aspartate showing a more pronounced increase under glucose deprivation. Aspartate is an important metabolic intermediate that supports anabolic processes and contributes to the maintenance of cellular redox homeostasis [26]. In addition, aspartate serves as a key precursor for pyrimidine biosynthesis, a pathway that is important for proliferating cells [27]. Our finding was independently validated using an aspartate assay kit in both MCF-7 and HEK293 cells. Importantly, exogenous aspartate was sufficient to suppress AMPK pathway activation and increase ATP/AMP levels under glucose deprivation, whereas reducing intracellular aspartate with AOA restored AMPK phosphorylation and partially normalized ATP/AMP levels. Add-back experiments further strengthened this link, as exogenous aspartate reversed the effects of AOA on intracellular aspartate levels, AMPK signaling, and ATP/AMP status. Together, these findings support the idea that abnormal aspartate accumulation is not merely associated with TRIM24 deficiency, but also contributes to the altered response to glucose deprivation. Although our data show that TRIM24 deficiency is associated with abnormal intracellular aspartate accumulation and that elevated aspartate functionally contributes to impaired AMPK pathway activation under glucose deprivation, the precise upstream mechanism by which loss of TRIM24 leads to aspartate accumulation remains to be determined. Potentially, this may involve altered regulation of aspartate production, utilization, catabolism, or compartmental flux during glucose deprivation, although these possibilities were not directly addressed in the present study.

At first glance, the observation that TRIM24-deficient cells maintain a relatively higher ATP/AMP ratio yet exhibit poorer survival under glucose deprivation may appear paradoxical. However, under glucose deprivation, both control and TRIM24-deficient cells showed a decline in ATP/AMP ratio, and the key difference was that this decrease was more pronounced in control cells. In our model, abnormal aspartate accumulation may partially sustain ATP/AMP levels and thereby blunt energy-stress sensing, preventing full activation of AMPK and its downstream adaptive responses. In this context, a relatively higher ATP/AMP ratio does not necessarily indicate a more favorable long-term metabolic state; instead, it may reflect insufficient activation of the signaling program required for adaptation to prolonged glucose limitation. In addition, ATP/AMP measurements obtained at the population level may not fully capture changes in absolute adenine nucleotide pools, temporal dynamics, or cell-to-cell heterogeneity. Accordingly, ATP/AMP measurements should be interpreted as one component of the energy-stress response rather than a comprehensive surrogate for global metabolic state. These factors may also contribute to the apparent divergence between ATP/AMP status and long-term survival.

These observations are broadly consistent with the model proposed by Zhu and colleagues, in which aspartate was shown to sustain ATP/AMP levels during starvation, attenuate AMPK activation, and impair adaptation to nutrient stress [22]. More broadly, this is also in line with previous studies indicating that aspartate is not only a biosynthetic metabolite, but can also influence stress adaptation through its impact on energy metabolism and signaling outputs. Our results extend this concept by suggesting that TRIM24 deficiency represents a cellular context in which abnormal aspartate accumulation becomes linked to defective AMPK pathway activation during glucose deprivation. At the same time, our data do not indicate that aspartate is the sole determinant of this phenotype. Rather, they support a model in which aspartate is an important mediator of the defective metabolic adaptation associated with TRIM24 deficiency during glucose deprivation.

An additional consideration is the metabolomic platform used in this study. We chose NMR-based metabolomic analysis because it provides robust reproducibility and relatively direct quantitative comparison across samples, which is advantageous for detecting consistent changes in relatively abundant metabolites under defined experimental conditions. Compared with mass spectrometry-based metabolomics, NMR generally has lower analytical sensitivity and more limited metabolite coverage, especially for low-abundance metabolites. Thus, our NMR analysis was intended to highlight prominent and reproducible metabolic differences rather than to provide exhaustive metabolite coverage. In this context, the identification of aspartate as an altered metabolite was further supported by an independent aspartate assay and by functional perturbation experiments. Nevertheless, broader mass spectrometry-based metabolomic profiling in future studies may help identify additional metabolic changes associated with TRIM24 deficiency.

Several limitations should be acknowledged. First, although AOA provided a useful pharmacological approach to reduce intracellular aspartate, it inhibits multiple transaminases and is therefore not specific for aspartate metabolism. The GOT2 knockdown experiments provide orthogonal genetic support for the involvement of aspartate-related metabolism, but they do not exclude contributions from additional aminotransferase-dependent pathways. Second, the upstream mechanism by which TRIM24 regulates aspartate homeostasis during glucose deprivation remains incompletely defined. Future studies will be needed to determine whether TRIM24 influences aspartate production, utilization, or compartmental flux through transcriptional or post-transcriptional mechanisms. Third, the in vivo data should be interpreted with appropriate caution. The mouse experiments were performed in a constitutive whole-body Trim24 knockout model, and Trim24−/− mice showed lower baseline body weight, making it difficult to distinguish tissue-intrinsic effects from broader systemic consequences of TRIM24 deficiency. Thus, the mouse data are best viewed as supportive evidence consistent with the cell-based findings rather than definitive in vivo mechanistic proof.

Finally, while our metabolomic analysis identified aspartate as a prominent altered metabolite under glucose deprivation, other metabolic changes may also contribute to the phenotype associated with TRIM24 deficiency. Future studies will be needed to define how TRIM24 regulates metabolic remodeling under nutrient stress and to determine the broader relevance of this pathway in physiological and pathological settings.

## Data Availability

The data presented in this study are available from the corresponding author upon reasonable request.

## Supplementary Materials

The following supporting information can be downloaded at: https://www.mdpi.com/article/10.3390/cimb48040403/s1.

## Abbreviations

The following abbreviations are used in this manuscript:

TRIM24	Tripartite motif-containing 24
TIF1α	Transcription intermediary factor 1α
AMPK	AMP-activated protein kinase
ACC	Acetyl-CoA carboxylase
ULK1	Unc-51-like kinase 1
AICAR	5-Aminoimidazole-4-carboxamide-1-β-D-ribofuranoside
AOA	Aminooxyacetic acid
GOT2	Glutamic-oxaloacetic transaminase 2
ATP	Adenosine triphosphate
AMP	Adenosine monophosphate
OPLS-DA	Orthogonal partial least squares discriminant analysis

## References

[1] Garcia D., Shaw R.J. (2017). AMPK: Mechanisms of Cellular Energy Sensing and Restoration of Metabolic Balance. Mol. Cell.

[2] Levy T., Voeltzke K., Hruby L., Alasad K., Bas Z., Snaebjornsson M., Marciano R., Scharov K., Planque M., Vriens K. (2024). mTORC1 regulates cell survival under glucose starvation through 4EBP1/2-mediated translational reprogramming of fatty acid metabolism. Nat. Commun..

[3] Scholtes C., Giguere V. (2022). Transcriptional control of energy metabolism by nuclear receptors. Nat. Rev. Mol. Cell Biol..

[4] Lee W.C., Guntur A.R., Long F., Rosen C.J. (2017). Energy Metabolism of the Osteoblast: Implications for Osteoporosis. Endocr. Rev..

[5] Liu H., Wang S., Wang J., Guo X., Song Y., Fu K., Gao Z., Liu D., He W., Yang L.L. (2025). Energy metabolism in health and diseases. Signal Transduct. Target. Ther..

[6] Korczowska-Lacka I., Hurla M., Banaszek N., Kobylarek D., Szymanowicz O., Kozubski W., Dorszewska J. (2023). Selected Biomarkers of Oxidative Stress and Energy Metabolism Disorders in Neurological Diseases. Mol. Neurobiol..

[7] Park J.M., Lee D.H., Kim D.H. (2023). Redefining the role of AMPK in autophagy and the energy stress response. Nat. Commun..

[8] Belanger M., Allaman I., Magistretti P.J. (2011). Brain energy metabolism: Focus on astrocyte-neuron metabolic cooperation. Cell Metab..

[9] Lin S.C., Hardie D.G. (2018). AMPK: Sensing Glucose as well as Cellular Energy Status. Cell Metab..

[10] Gowans G.J., Hawley S.A., Ross F.A., Hardie D.G. (2013). AMP is a true physiological regulator of AMP-activated protein kinase by both allosteric activation and enhancing net phosphorylation. Cell Metab..

[11] Steinberg G.R., Hardie D.G. (2023). New insights into activation and function of the AMPK. Nat. Rev. Mol. Cell Biol..

[12] Jeon S.M. (2016). Regulation and function of AMPK in physiology and diseases. Exp. Mol. Med..

[13] Malik N., Shaw R.J. (2025). The AMPK Pathway: Molecular Rejuvenation of Metabolism and Mitochondria. Annu. Rev. Cell Dev. Biol..

[14] Hatakeyama S. (2011). TRIM proteins and cancer. Nat. Rev. Cancer.

[15] Allton K., Jain A.K., Herz H.M., Tsai W.W., Jung S.Y., Qin J., Bergmann A., Johnson R.L., Barton M.C. (2009). Trim24 targets endogenous p53 for degradation. Proc. Natl. Acad. Sci. USA.

[16] Tsai W.W., Wang Z., Yiu T.T., Akdemir K.C., Xia W., Winter S., Tsai C.Y., Shi X., Schwarzer D., Plunkett W. (2010). TRIM24 links a non-canonical histone signature to breast cancer. Nature.

[17] Kim D., Bhargava R., Wang S.C., Tseng W.C., Lee D., Patel R., Oh S., Bowman R.W., Smith B.A., Kim M. (2025). TRIM24 directs replicative stress responses to maintain ALT telomeres via chromatin signaling. Mol. Cell.

[18] Groner A.C., Cato L., de Tribolet-Hardy J., Bernasocchi T., Janouskova H., Melchers D., Houtman R., Cato A.C.B., Tschopp P., Gu L. (2016). TRIM24 Is an Oncogenic Transcriptional Activator in Prostate Cancer. Cancer Cell.

[19] Zhang L.H., Yin Y.H., Chen H.Z., Feng S.Y., Liu J.L., Chen L., Fu W.L., Sun G.C., Yu X.G., Xu D.G. (2020). TRIM24 promotes stemness and invasiveness of glioblastoma cells via activating Sox2 expression. Neuro Oncol..

[20] Padrao N., Gregoricchio S., Eickhoff N., Dong J., Luzietti L., Bossi D., Severson T.M., Siefert J., Calcinotto A., Buluwela L. (2025). TRIM24 as a therapeutic target in endocrine treatment-resistant breast cancer. Proc. Natl. Acad. Sci. USA.

[21] Bao Y., Qian C., Liu M.Y., Jiang F., Jiang X., Liu H., Zhang Z., Sun F., Fu N., Hou Z. (2021). PRKAA/AMPKalpha phosphorylation switches the role of RASAL2 from a suppressor to an activator of autophagy. Autophagy.

[22] Mei X., Guo Y., Xie Z., Zhong Y., Wu X., Xu D., Li Y., Liu N., Zhu Z.J. (2021). RIPK1 regulates starvation resistance by modulating aspartate catabolism. Nat. Commun..

[23] Xu T., Stewart K.M., Wang X., Liu K., Xie M., Ryu J.K., Li K., Ma T., Wang H., Ni L. (2017). Metabolic control of T(H)17 and induced T(reg) cell balance by an epigenetic mechanism. Nature.

[24] Zhang L.H., Yin A.A., Cheng J.X., Huang H.Y., Li X.M., Zhang Y.Q., Han N., Zhang X. (2015). TRIM24 promotes glioma progression and enhances chemoresistance through activation of the PI3K/Akt signaling pathway. Oncogene.

[25] Chen S., Lin J., Yang Z., Wang Y., Wang Q., Wang D., Qu Y., Lin Q., Liu J., Yan S. (2026). TRIM24-mediated K27-linked ubiquitination of ULK1 alleviates energy stress-induced autophagy and promote prostate cancer growth in the context of SPOP mutation. Cell Death Differ..

[26] Birsoy K., Wang T., Chen W.W., Freinkman E., Abu-Remaileh M., Sabatini D.M. (2015). An Essential Role of the Mitochondrial Electron Transport Chain in Cell Proliferation Is to Enable Aspartate Synthesis. Cell.

[27] Sullivan L.B., Gui D.Y., Hosios A.M., Bush L.N., Freinkman E., Vander Heiden M.G. (2015). Supporting Aspartate Biosynthesis Is an Essential Function of Respiration in Proliferating Cells. Cell.

