# Supplementary material for: TRIM24 Regulates Adaptation to Glucose Deprivation in Association with Aspartate Accumulation and Impaired AMPK Signaling
Source: Curr Issues Mol Biol. 2026 Apr 14;48(4):403. doi: 10.3390/cimb48040403 (PMC13114581; doi:10.3390/cimb48040403)
Supplement: Supplementary file 1 [file cimb-48-00403-s001.zip › supplemental figures and legends.pdf]

Supplementary Information

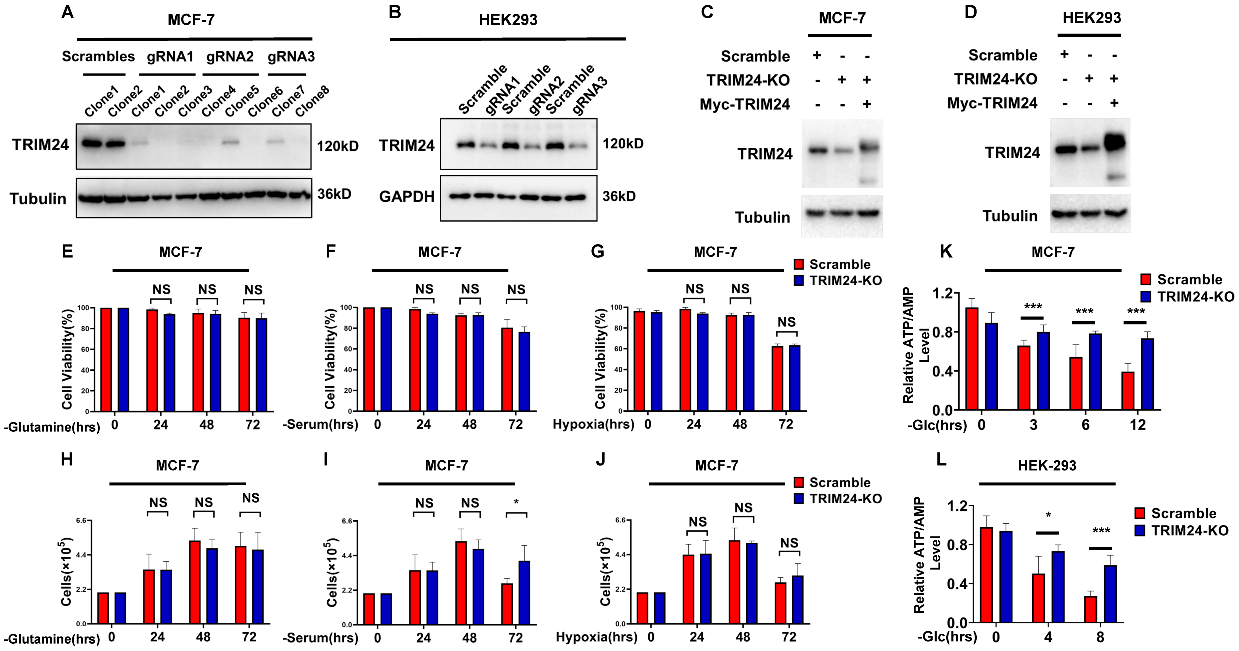

Figure S1. Validation of TRIM24-deficient MCF-7 clones and HEK293 mixed populations, TRIM24 re-expression, and characterization of cellular responses to different stress conditions.

(A) Immunoblot analysis of TRIM24 expression in MCF-7 single-cell-derived clones. The first two lanes show representative Scramble single-cell clones. TRIM24-targeted clones derived from gRNA1 (Clone1, Clone2, Clone3), gRNA2 (Clone4, Clone5, Clone6), and gRNA3 (Clone7, Clone8) are shown. Tubulin was used as a loading control.

(B) Immunoblot analysis of TRIM24 expression in HEK293 mixed populations generated using gRNA1, gRNA2, or gRNA3, together with corresponding Scramble control populations. All subsequent HEK293 experiments were performed using the gRNA1-derived mixed population. GAPDH was used as a loading control.

(C) Immunoblot analysis confirming re-expression of Myc-TRIM24 in established TRIM24-KO mixed MCF-7 cell populations used for the rescue experiment shown in Figure 1F. Tubulin was used as a loading control.

(D) Immunoblot analysis confirming re-expression of Myc-TRIM24 in established TRIM24-KO mixed HEK293 cell populations used for the rescue experiment shown in Figure 1G. Tubulin was used as a loading control.

(E) Cell viability of Scramble and TRIM24-KO MCF-7 cells cultured in glutamine-free medium for the indicated times (0, 24, 48, and 72 h). Cell viability was determined by

trypan blue staining and calculated as the percentage of viable cells relative to total cells.
(F) Cell viability of Scramble and TRIM24-KO MCF-7 cells cultured in serum-free medium for the indicated times (0, 24, 48, and 72 h). Cell viability was determined by trypan blue staining and calculated as the percentage of viable cells relative to total cells.
(G) Cell viability of Scramble and TRIM24-KO MCF-7 cells cultured under hypoxic conditions for the indicated times (0, 24, 48, and 72 h). Cell viability was determined by trypan blue staining and calculated as the percentage of viable cells relative to total cells.
(H) Viable cell numbers of Scramble and TRIM24-KO MCF-7 cells cultured in glutamine-free medium for the indicated times. Cell numbers were determined by trypan blue staining followed by counting of all viable cells.
(I) Viable cell numbers of Scramble and TRIM24-KO MCF-7 cells cultured in serum-free medium for the indicated times. Cell numbers were determined by trypan blue staining followed by counting of all viable cells.
(J) Viable cell numbers of Scramble and TRIM24-KO MCF-7 cells cultured under hypoxic conditions for the indicated times. Cell numbers were determined by trypan blue staining followed by counting of all viable cells.
(K) Relative ATP/AMP levels in Scramble and TRIM24-KO MCF-7 cells cultured in glucose-free medium for the indicated times (0, 3, 6, and 12 h).
(L) Relative ATP/AMP levels in Scramble and TRIM24-KO HEK293 cells cultured in glucose-free medium for the indicated times (0, 4, and 8 h).

Data are presented as mean ± SD from three independent experiments (n = 3). NS, not significant; * $p < 0.05$; *** $p < 0.001$.

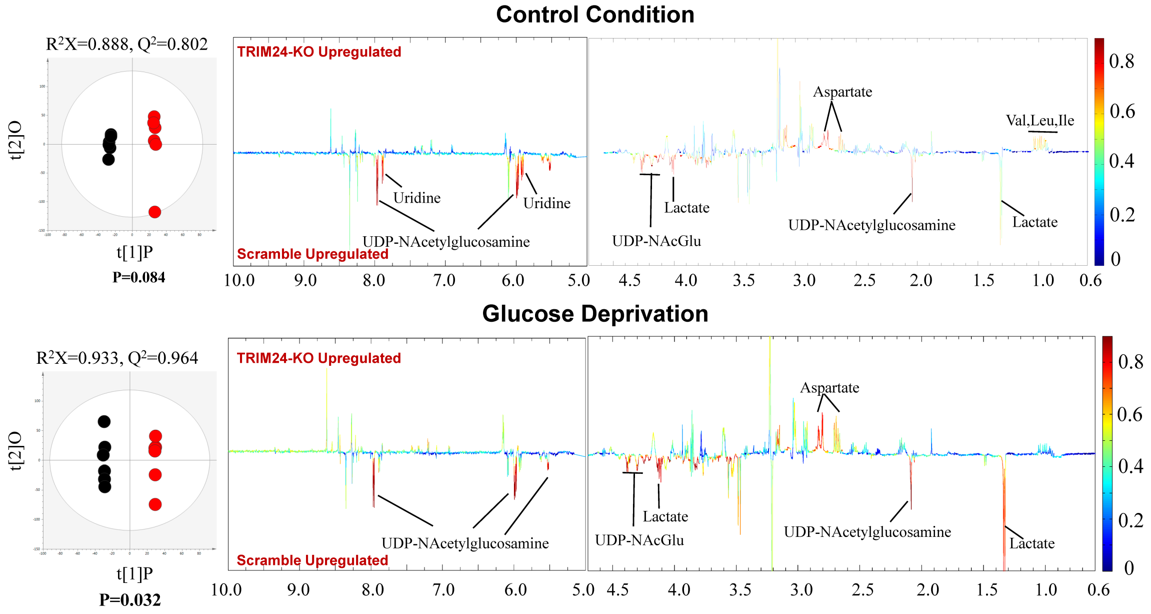

Figure S2. NMR-based metabolomic analysis of metabolic alterations associated with TRIM24 deficiency in MCF-7 cells under control and glucose deprivation conditions. Representative OPLS-DA score plots and corresponding differential ^{1}H NMR spectra comparing Scramble and TRIM24-KO MCF-7 cells cultured in normal medium or in glucose-free medium for 12 h. The upper panel shows the analysis under control conditions, and the lower panel shows the analysis after glucose deprivation. In the OPLS-DA score plots, t[1]P represents the first predictive component and t[2]O represents the second orthogonal component. R^2X indicates the proportion of variation in the metabolite data matrix explained by the model, and Q^2 indicates the predictive ability of the model estimated by cross-validation. In the differential ^{1}H NMR spectra, the x-axis represents chemical shift (ppm), and the y-axis represents relative spectral intensity. Signals displayed above the baseline indicate metabolites increased in TRIM24-KO cells, whereas signals displayed below the baseline indicate metabolites increased in Scramble cells. Metabolites were assigned by comparison with reference

databases together with spectral interpretation, and representative differential metabolites are indicated.

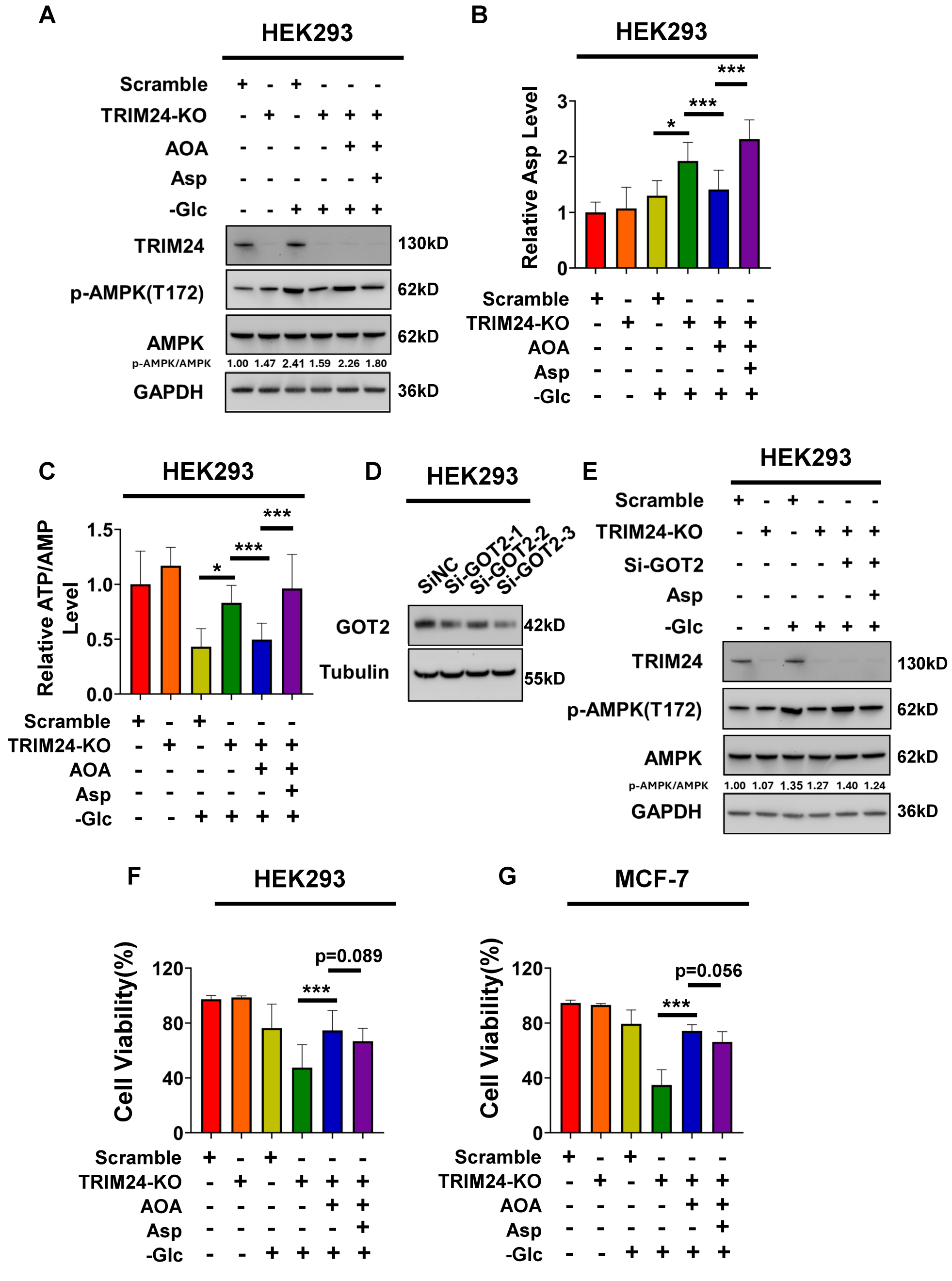

Figure S3. Aspartate modulation and GOT2 knockdown alter glucose deprivation responses in TRIM24-deficient HEK293 cells.

(A) Immunoblot analysis of phospho-AMPK (Thr172), total AMPK, and GAPDH in Scramble and TRIM24-KO HEK293 cells cultured under control conditions, in glucose-free medium alone, in glucose-free medium supplemented with AOA (750 μM), or in

glucose-free medium supplemented with AOA (750 μM) and aspartate (400 μM). GAPDH was used as a loading control. Densitometric values of p-AMPK/AMPK are indicated below the blots.

(B) Intracellular aspartate levels in Scramble and TRIM24-KO HEK293 cells cultured under control conditions, in glucose-free medium alone, in glucose-free medium supplemented with AOA (750 μM), or in glucose-free medium supplemented with AOA (750 μM) and aspartate (400 μM), as determined using an aspartate assay kit.

(C) Relative ATP/AMP levels in Scramble and TRIM24-KO HEK293 cells cultured under control conditions, in glucose-free medium alone, in glucose-free medium supplemented with AOA (750 μM), or in glucose-free medium supplemented with AOA (750 μM) and aspartate (400 μM).

(D) Immunoblot analysis of GOT2 expression in HEK293 cells transfected with three independent GOT2 siRNAs. Tubulin was used as a loading control.

(E) Immunoblot analysis of phospho-AMPK (Thr172), total AMPK, and GAPDH in Scramble and TRIM24-KO HEK293 cells cultured under control conditions, in glucose-free medium alone, or in glucose-free medium following GOT2 knockdown in the absence or presence of aspartate (400 μM). GAPDH was used as a loading control. Densitometric values of p-AMPK/AMPK are indicated below the blots.

(F) Cell viability of Scramble and TRIM24-KO HEK293 cells cultured under control conditions, in glucose-free medium alone, in glucose-free medium supplemented with AOA (750 μM), or in glucose-free medium supplemented with AOA (750 μM) and aspartate (400 μM). Cell viability was determined by trypan blue staining and calculated as the percentage of viable cells relative to total cells.

(G) Cell viability of Scramble and TRIM24-KO MCF-7 cells cultured under control conditions, in glucose-free medium alone, in glucose-free medium supplemented with AOA (500 μM), or in glucose-free medium supplemented with AOA (500 μM) and aspartate (400 μM). Cell viability was determined by trypan blue staining and calculated as the percentage of viable cells relative to total cells.

Data are presented as mean ± SD from three independent experiments (n = 3). ** $p < 0.01$; *** $p < 0.001$.

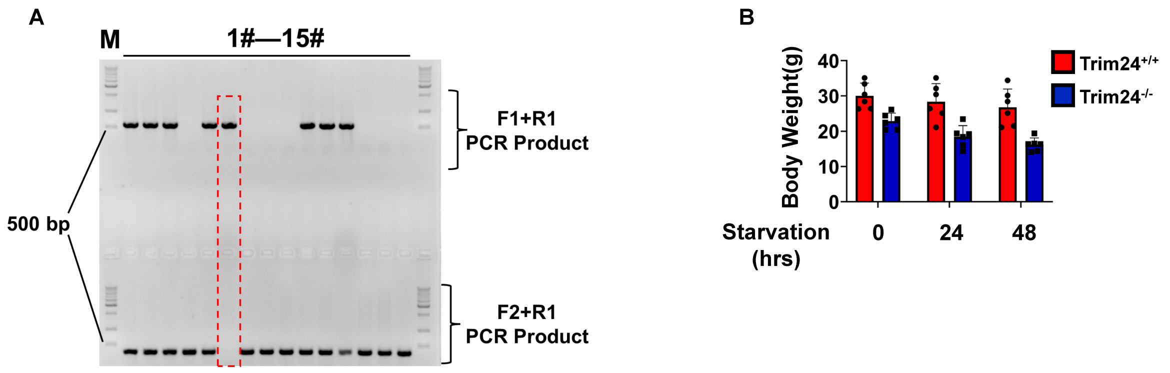

Figure S4. Genotyping and body weight analysis of Trim24 knockout mice.
(A) Representative genotyping results of Trim24 knockout mice. Mouse tail genomic DNA was analyzed using two PCR reactions. The F1/R1 primer pair detected the deleted Trim24 allele, whereas the F2/R1 primer pair detected the intact WT allele. Samples positive for the F1/R1 PCR product but negative for the F2/R1 PCR product were identified as homozygous Trim24 knockout mice. Samples positive for both PCR products were classified as heterozygous mice, whereas samples negative for the F1/R1 product but positive for the F2/R1 product were classified as WT mice. The dashed box indicates a representative homozygous Trim24 knockout sample.
(B) Body weight of Trim24+/+ and Trim24−/− mice measured at the indicated time points during 48 h starvation. Data are presented as mean ± SD (n = 6).
